# Head-to-Head Comparison of Oxidative Stress Biomarkers for All-Cause Mortality in Hemodialysis Patients

**DOI:** 10.3390/antiox11101975

**Published:** 2022-10-02

**Authors:** Jiao Zuo, Lyubov Chaykovska, Chang Chu, Xin Chen, Ahmed A. Hasan, Bernhard K. Krämer, Martin Tepel, Berthold Hocher

**Affiliations:** 1 Fifth Department of Medicine (Nephrology/Endocrinology/Rheumatology), University Medical Centre Mannheim, University of Heidelberg, 68167 Mannheim, Germany; 2 Department of Nephrology, Charite University Berlin, 13353 Berlin, Germany; 3 European Center for Angioscience ECAS, Medical Faculty Mannheim, University of Heidelberg, 68167 Mannheim, Germany; 4 Department of Nephrology, Odense University Hospital, 5000 Odense, Denmark; 5 Institute of Medical Diagnostics, IMD Berlin-Potsdam, 12247 Berlin, Germany; 6 Reproductive and Genetic Hospital of CITIC-Xiangya, Changsha 410008, China

**Keywords:** maintenance hemodialysis, oxidative stress, all-cause mortality, carbonyl proteins, myeloperoxidase, advanced oxidation protein products, oxidized low-density lipoprotein

## Abstract

Oxidative stress (OS) presents even in the early chronic kidney disease (CKD) stage and is exacerbated in patients with end-stage renal disease (ESRD) undergoing maintenance hemodialysis (MHD). There is still a debate over the association between oxidative stress and mortality. Our study aims to compare head-to-head the prognostic value of different oxidative markers for all-cause mortality in hemodialysis (HD) patients. We thus enrolled 347 patients on HD in this prospective study. Four OS biomarkers were measured (carbonyl proteins, myeloperoxidase (MPO), advanced oxidation protein products (AOPPs), and oxidized low-density lipoprotein (ox-LDL)). During the 60-month follow-up period, 9 patients have been lost to follow-up and 168 (48.4%) patients died. Concerning the oxidative stress (ox-stress) byproducts, carbonyl proteins were lower in survivors (105.40 ng/mL (IQR 81.30–147.85) versus 129.65 ng/mL (IQR 93.20–180.33); *p* < 0.001), with similar results for male patients (103.70 ng/mL (IQR 76.90–153.33) versus 134.55 ng/mL (IQR 93.95–178.68); *p* = 0.0014). However, there are no significant differences in MPO, AOPP, and ox-LDL between the two groups. Kaplan–Meier survival analysis indicated that patients in the higher carbonyl proteins concentration (>117.85 ng/mL group) had a significantly lower survival rate (log-rank test, *p* < 0.001). Univariate Cox regression analysis showed a positive correlation between carbonyl proteins and all-cause mortality in the higher and lower halves. Even after adjustment for conventional risk factors, it remained a statistically significant predictor of an increased risk of death in MHD. Univariate Cox regression analysis of MPO showed that continuous MPO and Log MPO were significantly associated with all-cause mortality, except for binary MPO (divided according to the median of MPO). Multivariate Cox analysis for MPO showed that the mortality prediction remains significant after adjusting for multiple factors. In conclusion, not all ox-stress biomarkers predict all-cause mortality in HD patients to a similar extent. In the present study, carbonyl proteins and MPO are independent predictors of all-cause mortality in HD patients, whereas AOPPs and oxLDL are clearly not associated with all-cause mortality in HD patients.

## 1. Introduction

End-stage renal disease (ESRD) patients are highly prone to acute-phase inflammation and oxidative stress, both linked with cardiovascular mortality and morbidity [1,2,3,4]. Additionally, maintenance dialysis patients have an excessively high risk of cardiovascular morbidity and mortality; even after adjustment, cardiovascular mortality has been reported to be 10 to 20-fold higher than in the general population [5]. The development of long-term complications such as amyloidosis, atherosclerosis, and cardiovascular disease (CVD) in hemodialysis (HD) patients may be influenced by oxidative stress, which may act synergistically with inflammation [4,6,7]. There is increasing recognition that oxidative stress is an important metabolic component of ESRD [6].

The imbalance between the generation of oxidant compounds and the defense mechanisms against them causes oxidative stress, described as tissue damage [7], which leads to a greater risk of atherosclerosis and b2-microglobulin amyloidosis, as well as significant oxidative stress in ESRD patients [6]. Oxidatively modified amino acids and plasma proteins can be important in vivo oxidative stress biomarkers [8]. The half-life of oxidants is only seconds, making them highly reactive compounds. Due to this, it is generally not possible to determine them in vivo. Unlike proteins, carbohydrates, and nucleic acids, oxidant-modified lipids have lifetimes ranging from hours to weeks, making them ideal indicators of oxidant stress [9].

The available studies have shown different results regarding the predictive role of different oxidative biomarkers for all-cause mortality. In this study, we aimed to assess the prognostic value of four different oxidative stress biomarkers (carbonyl proteins, myeloperoxidase (MPO), advanced oxidation protein products (AOPPs), and oxidized LDL (oxLDL)) for all-cause mortality in HD patients.

## 2. Materials and Methods

### 2.1. Study Population

In our study, we recruited 347 patients on stable hemodialysis from two dialysis centers associated with our inpatient facility at the Campus Charité Mitte (KfH Dialysezentrum-Neukölln, Berlin, Germany, and KfH Dialysezentrum-Moabit, Berlin, Germany). Local ethics committees approved this study (approval number: S-20090061), and informed consent was obtained from all study participants.

Hemodialysis with standard bicarbonate dialysis with biocompatible membranes was administered three to four times per week to all patients. Dialysate flow rates were 500 mL/min and blood flow rates were 250–300 mL/min. All patients had a functioning permanent access. The study excluded patients with malignancies, active infections, pregnancy, or unwillingness to participate. Every patient had a functional permanent access device. A 60-month follow-up period documented all-cause deaths. Patients who received a transplant were censored at the time of transplantation.

### 2.2. Assays

At the study entrance, blood samples were collected before each session of hemodialysis and the blood was drawn on a fasting state at the morning. Routine blood tests (hemoglobin, ferritin, transferrin, fasting blood glucose, creatinine, potassium, calcium, phosphorus, iPTH, *n*-ox PTH, albumin, BUN, LDL, HDL, hsCRP) were assessed by standardized methods in the clinical laboratory. The plasma biomarkers were analyzed using a sandwich enzyme immunoassay: Myeloperoxidase (MPO) [K6631B, in vitro determination of Myeloperoxidase in serum and plasma (ELISA), Immundiagnostik, AG, Bensheim, Germany], advanced oxidation protein products (AOPPs) [KR7811W, in vitro determination of Advanced oxidation protein products (AOPPs) in EDTA plasma (Photometric), Immundiagnostik, AG, Bensheim, Germany], oxidized low-density lipoprotein (ox-LDL) [K7810, in vitro determination of ox-LDL (ELISA), Immundiagnostik, AG, Bensheim, Germany], and Carbonyl proteins concentrations [K7870, in vitro determination of protein-bound carbonyls in human serum and plasma (ELISA), Immundiagnostik, AG, Bensheim, Germany] according to manufacturer instructions.

### 2.3. Statistical Analysis

Statistical significance was defined as *p* < 0.05. All analysis was performed using SPSS version 25.0 (IBM, Armonk, NY, USA). Descriptive variables are expressed as medians (interquartile ranges) or numbers (percentages). The Mann–Whitney U test was performed to determine the differences between the survivors and non-survivors. Cumulative survival curves were performed using the Kaplan–Meier method stratified by the median (lower and higher than values), and the log-rank test was used to compare groups’ survival. After conducting one-way regression analysis, those with *p* values less than 0.1 were included in the final multi-factor regression equation. Among them, iPTH and noxPTH interacted with each other in regression analysis, while *n*-oxPTH may better reflect the hormonal function [10], so noxPTH was selected to be included in the regression equation. The analysis of the simultaneous associations between risk factors and survival time was performed using the multivariate Cox regression analysis to control for possible confounding factors. Hazard ratios (HR) and their 95% confidence intervals (CI) were calculated. According to univariate Cox Regression results, we created three models for multivariate Cox regression analysis. Model A was an adjustment for demographics (age, hypertension, and CVD); Model B was an adjustment for clinical parameters (serum creatinine, transferrin, phosphorus, *n*-oxPTH, albumin); Model C was an adjustment for the risk factors in both model A and model B.

## 3. Results

**Figure 1:**
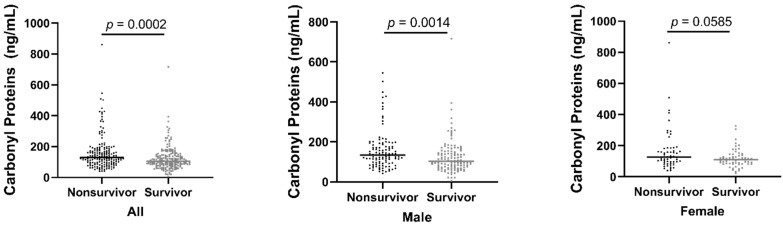
Plots of serum carbonyl proteins concentrations. Median serum carbonyl proteins were significantly lower in the survivors than the non-survivors using the Mann–Whitney U test.

A total of 347 HD patients were included in this study; the median age was 66 years (IQR 56–75). There were 229 male patients, 117 female patients, and 1 patient with no sex indicated. In total, 130 patients had diabetes mellitus (DM) and 161 had a history of CVD. More than three-quarters of patients had hypertension (77.5%). According to the outcome, we divided the HD patients into two groups: survivors and non-survivors. Demographic and clinical data within each group are presented in Table 1. During the 60-month follow-up period, 9 patients (including 1 patient of unknown sex) were lost to follow-up, and 168 (48.4%) patients died. Among the 347 HD patients, survivors were younger, had a lower prevalence of DM and CVD, and had significantly lower hsCRP concentrations, while having higher transferrin, fasting blood glucose, intact parathyroid hormone (iPTH), non-oxidized parathyroid hormone, serum albumin, and LDL compared to non-survivors. Concerning the ox-stress byproducts, carbonyl proteins were lower in survivors (105.40 ng/mL (IQR 81.30–147.85) versus 129.65 ng/mL (IQR 93.20–180.33); *p* < 0.001) (Figure 1), and among male survivors, this trend continues (103.70 ng/mL (IQR 76.90–153.33) versus 134.55 ng/mL (IQR 93.95–178.68); *p* = 0.0014) (Figure 1). However, there are no significant differences in MPO, AOPPs, and ox-LDL between the two groups (Table 1; Supplementary Figure S1).

**Figure 2:**
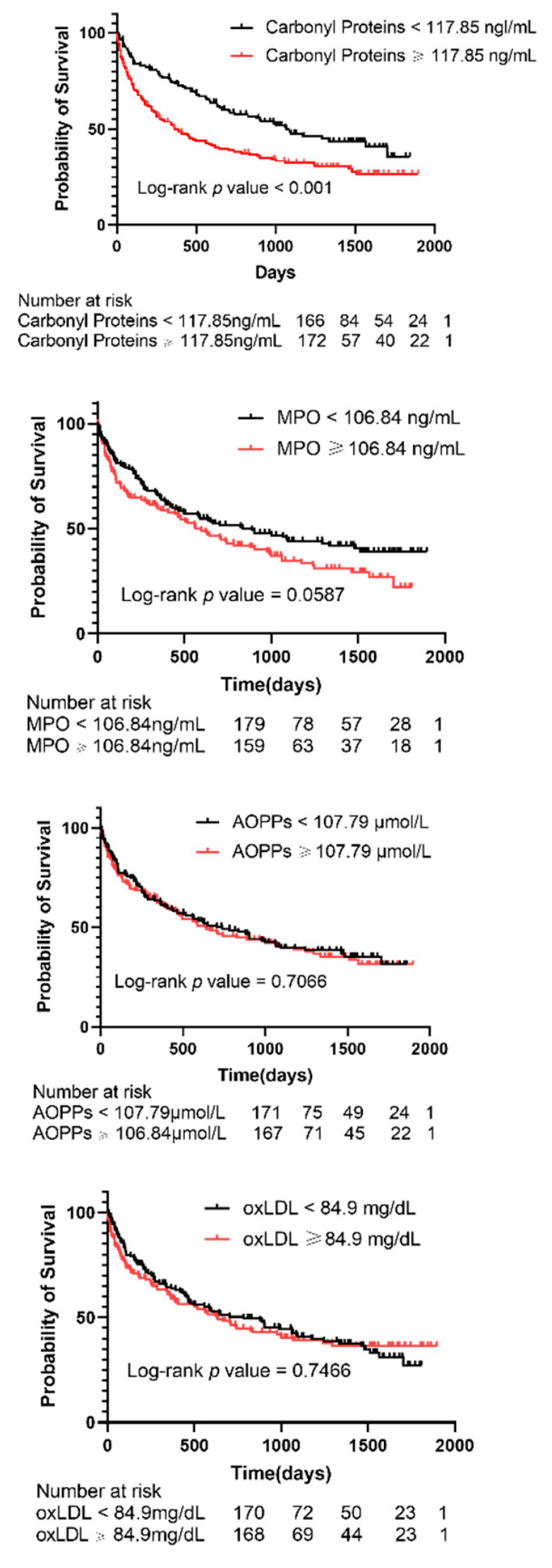
Kaplan–Meier survival curves for all-cause mortality. Patients were divided according to the median values of variables. Abbreviations: MPO: Myeloperoxidase; AOPPs: Advanced oxidation protein products; ox-LDL: Oxidized low-density lipoprotein.

Kaplan–Meier curves for all-cause mortality according to the median of each of four ox-stress byproduct concentrations at the baseline are presented in Figure 2. It revealed that the lower carbonyl proteins concentration group (<117.85 ng/mL) had a significantly higher survival rate (log-rank test, *p* < 0.001) in this study cohort (Figure 2). AOPPs, MPO, and oxLDL did not show statistical significance.

Then, we performed univariate and multivariate Cox regression analysis. Univariate Cox’s proportional hazards regression analysis showed that age (HR = 1.062 CI 95% (1.047–1.077) *p* < 0.001), CVD (HR = 1.440 CI 95% (1.056–1.963) *p* = 0.021), transferrin (HR = 0.995 CI 95% (0.992–0.998) *p* = 0.002), creatinine (HR = 0.875 CI 95% (0.819–0.935) *p* < 0.001), phosphorus (HR = 0.771 CI 95% (0.595–1.000) *p* = 0.05), iPTH (HR = 0.998 CI 95% (0.997–1.000) *p* = 0.012), *n*-oxPTH (HR = 0.986 CI 95% (0.973–1.000) *p* = 0.043), albumin (HR = 0.663 CI 95% (0.515–0.855) *p* = 0.001), MPO (HR = 1.000 CI 95% (1.000–1.000) *p* < 0.001) and carbonyl proteins (HR = 1.002 CI 95% (1.001–1.003) *p* = 0.001) had a significant association with survival (Table 2). After adjustment for the conventional risk factors of HD patients in different models (as described in the Materials and Methods section), baseline concentrations of carbonyl proteins remained a statistically significant predictor of an increased risk of death (Table 3). Continuous MPO and Log MPO were significantly associated with all-cause mortality, except binary MPO (divided according to the median of MPO) (Table 4). The mortality prediction of MPO remained significant after adjusting for multiple factors.

## 4. Discussion

In this study, four biomarkers of oxidative stress are evaluated as predictors of long-term mortality among patients with HD. Substantial differences were seen regarding the predictive power of different oxidative stress biomarkers to predict all-cause mortality in patients on dialysis. Baseline carbonyl proteins were lower in survivors versus non-survivors (Figure 1), whereas baseline MPO, AOPPs, and oxLDL did not differ between survivors and non-survivors (Supplementary Figure S1). When performing Cox regression analysis considering confounding factors showed that both carbonyl proteins and MPO were independent predictors of all-cause mortality in HD patients. AOPPs and oxLDL, on the other hand, were not independently associated with all-cause mortality,

Proteins constitute 70% of the tissue and cell dry mass and proteins are a major target for damage/posttranslational modifications [11,12]. One of the most widely used stable biomarkers for detecting severe oxidative protein damage is carbonyl proteins, which have been found to remain elevated in the blood for up to 18 h [13]. As a sign of oxidative protein damage, protein carbonylation occurs when lysine, arginine, proline, and threonine residues are directly oxidized, and when reactive carbonyl species are produced from carbohydrate and lipid oxidation interact with dicarbonyl compounds directly [14]. The process of carbonylation is irreversible and antioxidant defenses cannot effectively reverse this modification [15,16]. It is thought that carbonylation negatively affects both protein function and cellular viability [17,18,19,20,21]. Additionally, carbonylation may lead to highly cytotoxic large protease-resistant protein aggregates [22]. The level of plasma carbonyl proteins is higher in hemodialysis patients than in healthy individuals [23,24]. Our study showed higher carbonyl proteins level in HD non-survivable patients. Carbonyl proteins were good predictors of all-cause mortality in dialysis patients even after adjustments for multiple risk factors. Our data are in good agreement with a recent study also performed in HD patients (Supplementary Table S1) [25]. It is hypothesized that contact with the dialysis filter activates neutrophils, likely increasing oxidative/carbonyl stress and inflammation following HD [26]. However, there is also one other study who did not show an effect of protein carbonylation on mortality. This study just analyzed 44 patients (Supplementary Table S1) [27]. The power was thus probably too low.

As a major component of leukocytes’ bactericidal arsenal, myeloperoxidase (MPO), a heme enzyme synthesized and secreted by neutrophils and monocytic cells, is an important source of Reactive Oxygen Species (ROS) [28]. At inflammation sites, MPO is a major catalyst for lipid peroxidation, a process crucial to atherogenesis [29,30,31,32,33,34,35]. Plasma MPO levels appear to be increased during HD due to oxidative stress as well [36]. Dialysis may increase MPO through leukocyte activation at the dialysis membrane, and the degree of MPO may depend on the biocompatibility of the dialysis membrane [37,38,39]. A study including 356 patients on maintenance dialysis showed that increased MPO levels were independently associated with an increased risk of death and that measuring MPO may be useful for diagnosing unrecognized clinical risks (Supplementary Table S1) [40]. MPO may predict long-term mortality in HD patients was also confirmed in a comparative study (Supplementary Table S1) [41]. However, in another 5-year follow-up study of dialysis patients, MPO did not show an independent ability to predict all-cause mortality (Supplementary Table S1) [42]. We found that MPO had limited value in predicting all-cause mortality in our cohort and, unadjusted baseline values were similar in survivors and non-survivors. Only after adjusting for demographic and clinical risk factors in multivariate Cox analysis and continuous univariate correlation analysis was independently associated with all-cause mortality. In addition, this hazard risk of binary transformed MPO for all-cause mortality lost significance.

As a result of oxidative damage, proteins can develop modifications in their spectroscopic characteristics called advanced oxidation protein products (AOPPs) [43]. The AOPPs also promotes the production of reactive oxygen species as a byproduct of oxidative damage [44]. In comparison to lipid peroxidation products, AOPPs are more accurate for the measurement of oxidative stress [43]. These proteins are highly elevated in HD patients [45]. In healthy individuals and HD patients, AOPPs has been implicated as a risk factor for atherosclerotic cardiovascular events [46]. An 8-year follow-up prospective study of 199 patients with ESRD on hemodialysis showed that AOPPs demonstrated a significant predictive impact in overall and cardiovascular survival (Supplementary Table S1) [47]. Additionally, a multi-center, prospective cohort study showed that elevated serum AOPP levels were associated with higher risk of all-cause mortality in Chinese maintenance HD patients (Supplementary Table S1) [48]. In our study, AOPPs were not found to be a predictor of mortality in HD patients. There was even no trend. Different results from AOPPs for predicting all-cause mortality could be explained by two factors: first, a higher probability and odds of death would be predicted for patients on ESRD dialysis with an 8-year follow-up; second, more than half of the patients in this 8-year follow-up study were women, whereas almost half of the patients in our study were males. However, our result was consistent with the 112 HD patients, 5.5-year follow-up study (Supplementary Table S1) [49].

Oxidized low-density lipoprotein (oxLDL), a form of LDL formed after oxidation of LDL, is necessary for macrophages to accumulate cholesterol [50]. The measurement of oxLDL may provide better predictability of atherosclerotic CVD in patients with HD than total serum LDL cholesterol [51], because the increased monocyte endothelial cell adhesion associated with high oxLDL may contribute to CVD development in chronic renal failure patients on dialysis through another mechanism that interferes with coagulation activation [52]. Some studies showed that HD patients have increased oxLDL [53,54,55]. In contrast, other studies have reported that the oxLDL levels of HD patients were similar to those of the general population [56,57,58,59], or even lower [60]. Although oxLDL levels were associated with stable coronary artery disease and acute coronary syndromes [61], in HD patients, the findings of the relationship between oxLDL and mortality are controversial. OxLDL has limited clinical value in identifying the risk of vascular complications in young HD patients [56], with no difference seen between CVD and non-CVD groups (Supplementary Table S1) [62], and there are also studies showing that oxLDL is not associated with coronary artery calcification in MHD patients [63]. In patients not receiving HD in the LURIC study, there was no correlation between oxLDL and mortality (Supplementary Table S1) [64]. Another prospective observational study showed that oxLDL and anti-oxLDL in HD patients are not associated with overall mortality or cardiovascular mortality [50]. Similarly, no association with all-cause mortality was found in our study. When LDL is highly oxidized, it becomes pro-apoptotic and fails to be recognized by the LDL receptor (LDLR) [65]. Alternatively, oxLDL is absorbed by macrophage scavenger receptors, causing macrophage foam cells to form. This causes oxLDL cannot last too long in circulation, perhaps that is why oxLDL was not correlated with all-cause mortality in HD patients [66,67].

This study is the first to make a head-to-head comparison of HD patients’ four ox-stress biomarkers (carbonyl proteins, MPO, AOPPs, and oxLDL) with all-cause mortality and clearly shows that carbonyl proteins are superior biomarkers of all-cause mortality in HD patients. MPO, on the other hand, seems to be a somewhat weaker all-cause mortality biomarker, while oxLDL and AOPPs seem to have no impact on all-cause mortality in HD patients. Our study hence may be a useful tool to select ox-stress biomarkers for clinical use.

Our study also has clearly limitations, first we just had data on all-cause mortality but not on cardiovascular mortality. Second, we had no information of the use of any anti-oxidative substances by our patients. However, in contrast to previous studies, we used the approach of comparing key biomarkers for oxidative stress that are widely used but have never been compared head-to-head.

## 5. Conclusions

In conclusion, not all ox-stress markers predict all-cause mortality in HD patients with equal power. In the present study, especially carbonyl proteins but also MPO were found to be independent predictors of all-cause mortality for HD patients’ however, AOPPs and oxLDL failed to predict all-cause mortality.

## Figures and Tables

**Table 1:** Clinical and biochemical characteristics of dialysis patients.

Characteristics	All (*n* = 347)	Survivors (*n* = 170)	Non-Survivors (*n* = 168)	*p*-Value
Age (years)	66.0 (56.0–75.0)	60.50 (49.00–69.00)	71.00 (66.00–78.00)	<0.001
Sex (M/F/Unknown)	229/117/1	114/56/0	110/58/0	0.759
Body mass index, kg/m^2^	24.40 (22.01–27.60)	24.20 (22.12–28.30)	24.57 (21.71–26.99)	0.541
Drinker, *n* (%)	62 (17.90%)	30 (17.60%)	32 (9.10%)	0.740
Smoker, *n* (%)	108 (31.10%)	54 (31.80%)	52 (14.80%)	0.872
Diabetes mellitus, *n* (%)	130 (37.50%)	55 (32.40%)	74 (21.10%)	0.027
Hypertension, *n* (%)	269 (77.50%)	134 (78.80%)	135 (38.50%)	0.727
Cardiovascular disease, *n* (%)	161 (46.40%)	82 (48.20%)	101 (28.80%)	<0.001
Dialysis vintage (days)	263.00 (31.00–1219.25)	221.00 (31.00–939.25)	351.00 (31.00–1461.00)	0.004
Dialysis dose (Kt/V)	1.04 (0.91–1.16)	1.03 (0.91–1.16)	1.04 (0.91–1.17)	0.749
Medication, *n* (%)				
RAAS inhibitors	88 (25.40%)	46 (27.1%)	41 (11.70%)	0.577
Beta-blockers	204 (58.8%)	116 (68.2%)	86 (24.50%)	0.001
Calcium channel blockers	104 (30.00%)	60 (35.3%)	43 (12.30%)	0.053
Erythropoietin	171 (49.30%)	82 (48.2%)	89 (25.40%)	0.414
Diuretics	194 (55.90%)	98 (57.6%)	95 (27.10%)	0.838
Hemoglobin (g/dL)	10.20 (9.10–11.63)	10.25 (9.00–11.67)	10.20 (9.20–11.70)	0.865
Ferritin (ng/mL)	532.00 (253.25–1125.88)	527.50 (225.00–1065.75)	532.00 (281.00–1235.00)	0.540
Transferrin (µg/mL)	138.00(106.00–173.00)	145.00 (121.00–173.50)	128.50 (99.00–172.25)	0.003
Fasting blood glucose (mg/dL)	108.00 (90.00–134.00)	114.50 (94.50–143.60)	104.00 (87.00–123.60)	0.006
Creatinine (mg/dL)	6.62 (4.23–8.34)	6.67 (4.15–8.53)	6.60 (4.23–7.96)	0.007
Potassium (mmol/L)	4.70 (4.10–5.28)	4.60 (4.00–5.30)	4.77 (4.21–5.26)	0.734
Calcium (mmol/L)	2.24 (2.10–2.40)	2.20 (2.09–2.40)	2.27 (2.10–2.47)	0.414
Phosphorus (mmol/L)	1.61 (1.19–2.10)	1.70 (1.22–2.12)	1.54 (1.11–2.06)	0.051
iPTH (ng/L)	49.90 (18.68–124.60)	68.19 (21.75–171.05)	39.76 (14.47–101.90)	0.003
*n*-ox PTH (ng/L)	5.86 (2.38–14.01)	7.18 (3.05–16.26)	4.99 (1.98–11.11)	0.003
Albumin (g/dL)	3.30 (2.90–3.70)	3.40 (3.05–3.80)	3.10 (2.80–3.60)	0.001
BUN (mg/dL)	195.12 (146.70–267.67)	201.05 (152.64–267.67)	189.63 (131.73–279.50)	0.822
LDL (mg/dL)	92.70 (72.20–121.20)	100.80 (75.05–127.40)	89.00 (70.70–112.00)	0.013
HDL (mg/dL)	39.90 (32.20–50.80)	38.60 (31.00–50.20)	42.30 (34.30–54.00)	0.435
hsCRP (mg/L)	2.60 (1.00–5.20)	2.30 (0.70–4.50)	2.80 (1.20–6.63)	0.006
MPO (ng/mL)	106.84 (67.71–188.38)	102.27 (67.37–176.37)	118.90 (69.46–199.24)	0.176
AOPPs (µmol/L)	107.79 (78.79–149.94)	109.99 (80.59–156.72)	107.57 (79.53–146.80)	0.588
ox-LDL (mg/dL)	84.90 (44.80–180.55)	87.55 (45.85–197.63)	83.10 (44.53–176.35)	0.779
Carbonyl proteins (ng/mL)	117.85 (84.73–163.18)	105.40 (81.30–147.85)	129.65 (93.20–180.33)	<0.001

Values are presented as median (IQR). Between groups (survivors versus non-survivors) comparisons were made using a nonparametric Mann–Whitney U test for continuous variables and the Chi-test for categorical variables. 1 patient who did not indicate sex showed in this table as unknown. Abbreviations: RAAS: Renin-Angiotensin-Aldosterone-System; iPTH: intact Parathyroid hormone; *n*-oxPTH: non-oxidized Parathyroid hormone; BUN: Blood urea nitrogen; LDL: Low-density lipoprotein; HDL: High-density lipoprotein; hsCRP: High sensitivity C-reactive protein; MPO: Myeloperoxidase; AOPPs: Advanced oxidation protein products; ox-LDL: Oxidized low-density lipoprotein.

**Table 2:** Cox regression univariate analysis, hazard ratio, and 95% confidence intervals for survival in HD patients.

Analyses	HR (95% CI)	*p*-Value
Age(years)	1.062 (1.047–1.077)	<0.001
Male/Female	0.981 (0.714–1.349)	0.908
Body mass index, kg/m^2^	0.992 (0.961–1.024)	0.615
Drinker, *n* (%)	1.108 (0.754–1.629)	0.601
Smoker, *n* (%)	0.879 (0.634–1.220)	0.441
Diabetes mellitus, *n* (%)	1.203 (0.887–1.632)	0.235
Hypertension, *n* (%)	0.723 (0.493–1.059)	0.096
Cardiovascular disease, *n* (%)	1.440 (1.056–1.963)	0.021
Dialysis vintage (days)	0.999869 (0.999716–1.000022)	0.093
Dialysis dose (Kt/V)	0.731 (0.377–1.417)	0.353
Hemoglobin (g/dL)	0.950 (0.868–1.038)	0.256
Ferritin (ng/mL)	1.000 (1.000–1.000)	0.846
Transferrin (µg/mL)	0.995 (0.992–0.998)	0.002
Fasting blood glucose (mg/dL)	0.999 (0.995–1.002)	0.449
Creatinine (mg/dL)	0.875 (0.819–0.935)	<0.001
Potassium (mmol/L)	0.895 (0.744–1.076)	0.238
Calcium (mmol/L)	0.832 (0.493–1.403)	0.490
Phosphorus (mmol/L)	0.771 (0.595–1.000)	0.0503
iPTH (ng/L)	0.998 (0.997–1.000)	0.012
*n*-ox PTH	0.986 (0.973–1.000)	0.043
Albumin (g/dL)	0.663 (0.515–0.855)	0.001
BUN (mg/dL)	1.000 (0.999–1.001)	0.479
LDL (mg/dL)	0.997 (0.993–1.002)	0.250
HDL (mg/dL)	1.005 (0.996–1.015)	0.302
hsCRP (mg/L)	1.018 (0.992–1.044)	0.179
MPO (ng/mL)	1.000035 (1.000020–1.000051)	<0.001
AOPPs (µmol/L)	1.001 (0.998–1.004)	0.445
ox-LDL (mg/dL)	1.000 (0.999–1.000)	0.451
Carbonyl proteins (ng/mL)	1.002 (1.001–1.003)	0.001

Abbreviations: iPTH: intact Parathyroid hormone; *n*-oxPTH: non-oxidized Parathyroid hormone; BUN: Blood urea nitrogen; LDL: Low-density lipoprotein; HDL: High-density lipoprotein; hsCRP: High sensitivity C-reactive protein; MPO: Myeloperoxidase; AOPPs: Advanced oxidation protein products; ox-LDL: Oxidized low-density lipoprotein.

**Table 3:** Cox regression univariate and multivariate analysis of carbonyl proteins, hazard ratio, and 95% confidence intervals for survival in HD patients.

Analyses	HR (95% CI)	*p*-Value
Univariate Cox regression		
Continuous Carbonyl proteins	1.002 (1.001–1.003)	0.001
Binary Carbonyl proteins	0.564 (0.414–0.767)	<0.001
Log Carbonyl proteins	3.162 (1.684–5.937)	<0.001
Multivariable Cox regression		
Model A	1.002 (1.001–1.004)	0.001
Model B	1.002 (1.000–1.003)	0.027
Model C	1.002 (1.000–1.004)	0.015

Binary carbonyl proteins were divided according to the median of carbonyl proteins (117.85 ng/mL). Model A was adjusted for age, hypertension, and CVD; Model B was adjusted for serum creatinine, transferrin, phosphorus; albumin, and *n*-oxPTH; Model C was adjusted for the above risk factors (Model A + Model B).

**Table 4:** Cox regression univariate and multivariate analysis of MPO, hazard ratio, and 95% confidence intervals for survival in HD patients.

Analyses	HR (95% CI)	*p*-Value
Univariate Cox regression		
Continuous MPO	1.000035 (1.000020–1.000051)	<0.001
Binary MPO	1.363 (0.998–1.862)	0.052
Log MPO	2.123 (1.394–3.234)	<0.001
Multivariable Cox regression		
Model A	1.000033 (1.000018–1.000049)	<0.001
Model B	1.000028 (1.000012–1.000044)	<0.001
Model C	1.000024 (1.000008–1.000040)	0.003

Binary MPO was divided according to the median of MPO (106.84 ng/mL). Model A was adjusted for age, hypertension, and CVD; Model B was adjusted for serum creatinine, transferrin, phosphorus; albumin, and *n*-oxPTH; Model C was adjusted for the above risk factors (Model A + Model B).

## Data Availability

The data presented in this study are available in the article and Supplementary Materials.

## Supplementary Materials

The following supporting information can be downloaded at https://www.mdpi.com/article/10.3390/antiox11101975/s1, Figure S1: Plots of serum MPO, AOPPs, oxLDL. Table S1: Clinical studies that examined the four OS markers in hemodialyzed patients. References [25,27,40,41,42,47,48,49,50,62,64] are cited in the Supplementary Materials.
