# Supplementary material for: Head-to-Head Comparison of Oxidative Stress Biomarkers for All-Cause Mortality in Hemodialysis Patients
Source: Antioxidants (Basel). 2022 Oct 2;11(10):1975. doi: 10.3390/antiox11101975 (PMC9598936; doi:10.3390/antiox11101975)
Supplement: Supplementary file 1 [file antioxidants-11-01975-s001.zip › antioxidants-1922176-supplementary.pdf]

Supplementary Table S1. Clinical studies that examined the four OS markers in hemodialyzed patients.

Study	Samples	Follow-up	OS marker	Results
Zhou, Chun et al.[1]	1394 Chinese HD patients	5.2 years	AOPPs	Elevated serum AOPP levels were associated with higher risk of all-cause mortality in Chinese maintenance HD patients.
Song, Young Rim et al.[2]	88 HD patients	5.2 years	Protein carbonyl	Multivariate analysis showed that the serum levels of protein carbonyl (Hazard ratio [HR] 2.37, 95% CI 1.02–5.55, $p = 0.036$) was independent determinants of all-cause mortality.
Suvakov, Sonja et al. [3]	199 HD patients	8 years	AOPPs, PAB (prooxidant-antioxidant balance), MDA (malondialdehyde), GSTM1, sVCAM-1/sICAM-1	AOPP showed prognostic potential in terms of cardiovascular survival.
Wagner, Sandra, et al.[4]	2773 patients	4 years	oxLDL	Not anymore after adjusting for apolipoprotein B level in AURORA and was not related to mortality in LURIC.
Rusu, Crina Claudia et al.[5]	44 HD patients	9 years	bound MDA (bMDA), Protein carbonyls, ceruloplasmin, nitric oxide	Only bMDA was related to survival.

Hsiao, Kuang-Chih et al. [6]	40 HD patients	5 years	Albumin, MMP-2/9 (matrix metalloproteinases), MPO, SDF-1	MPO didn't show significant value for predicting survival
Lee, Young-Ki, et al.[7]	69 HD patients	5 years	oxLDL and LPC (lysophosphatidylcholine)	OxLDL is not associated with an increased risk of CVD among a group of Korean hemodialysis patients.
Sevinc Ok, Ebru, et al. [8]	124 HD patients	3 years	ox-LDL, anti-oxLDL	Neither oxidized nor anti-oxidized low-density lipoprotein level is associated with atherosclerosis or mortality.
Wang, Angela Yee-Moon et al. [9]	236 PD patients	3 years	MPO	The first article showed MPO level provides significant independent and additional predictive value for long-term mortality and cardiovascular events in patients with ESRD on maintenance PD therapy
Pachaly, Maria A et al. [10]	112 HD patients	5.5 years	IL-6, AOPPs, pentosidine, homocysteine	According to the median of AOPPs, there were no significant differences in survival rate.
Kalantar-Zadeh, Kamyar et al. [11]	356 MHD patients	3 years	MPO	Serum MPO levels correlate with levels of markers of inflammation and prospective mortality risk.

Abbreviations: OS: oxidative stress; CVD: Cardiovascular disease; ESRD: end-stage renal disease; HD: hemodialysis; MHD: maintenance hemodialysis; MPO: Myeloperoxidase; AOPPs: Advanced oxidation protein products; ox-LDL: Oxidized low-density lipoprotein.

Supplementary Figure S1. Plots of serum MPO, AOPPs, oxLDL.

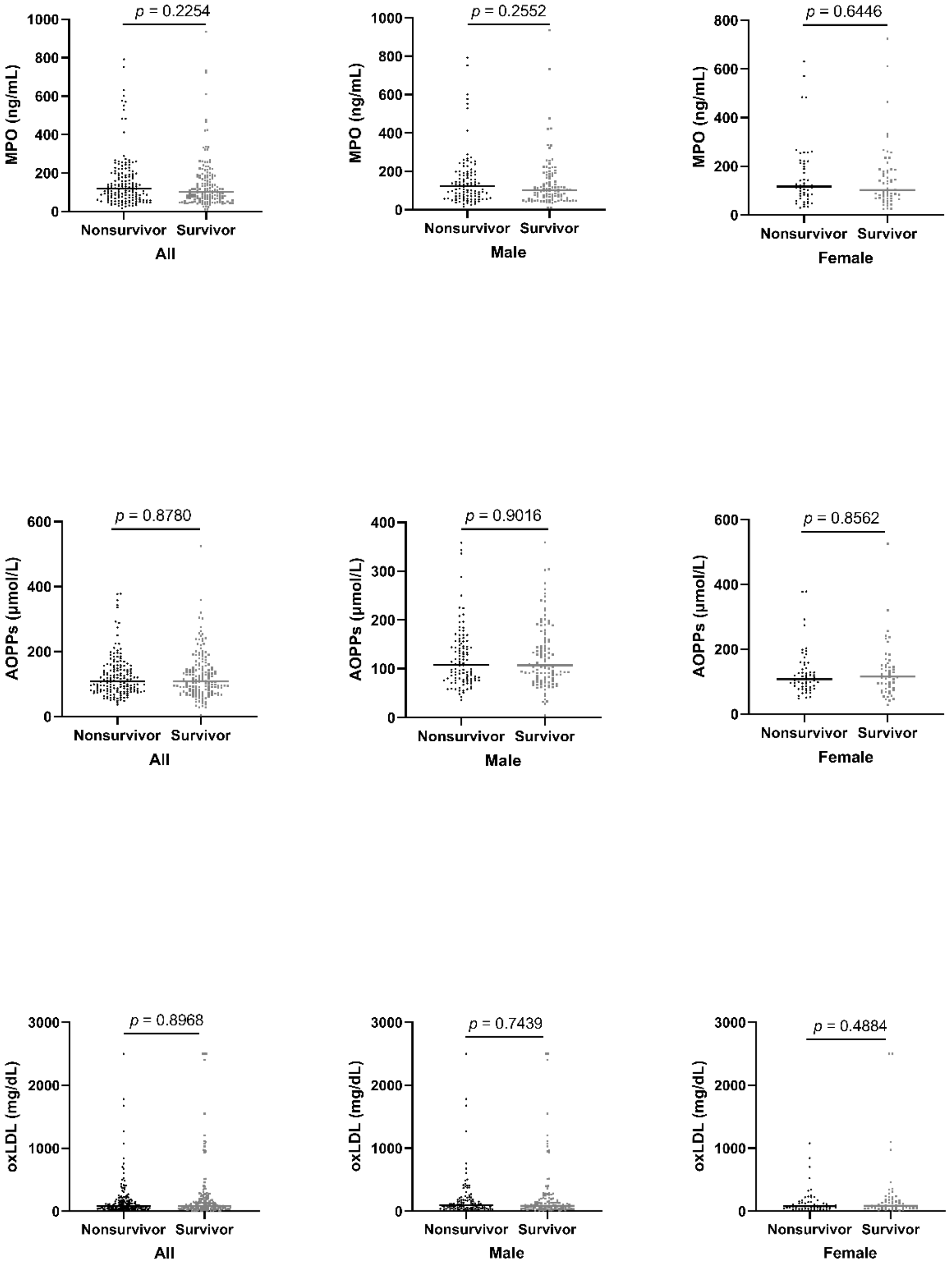

No significant differences in median MPO, AOPPs, and oxLDL were found between survivors and non-survivors using the Mann-Whitney U test.

Abbreviations: MPO: Myeloperoxidase; AOPPs: Advanced oxidation protein products; ox-LDL: Oxidized low-density lipoprotein.

Reference

1. Zhou, C., Y. Zhang, J. Chen, C. Mei, F. Xiong, W. Shi, W. Zhou, X. Liu, S. Sun, J. Tian, et al., Association between serum advanced oxidation protein products and mortality risk in maintenance hemodialysis patients. J Transl Med, 2021. 19(1): p. 284.
2. Song, Y.R., J.K. Kim, H.S. Lee, S.G. Kim, and E.K. Choi, Serum levels of protein carbonyl, a marker of oxidative stress, are associated with overhydration, sarcopenia and mortality in hemodialysis patients. BMC Nephrol, 2020. 21(1): p. 281.
3. Suvakov, S., D. Jerotic, T. Damjanovic, N. Milic, T. Pekmezovic, T. Djukic, Z. Jelic-Ivanovic, A. Savic Radojevic, M. Pljesa-Ercegovac, M. Matic, et al., Markers of Oxidative Stress and Endothelial Dysfunction Predict Haemodialysis Patients Survival. Am J Nephrol, 2019. 50(2): p. 115-125.
4. Wagner, S., M. Apetrii, Z.A. Massy, M.E. Kleber, G.E. Delgado, H. Scharnagel, W. März, M. Metzger, P. Rossignol, A. Jardine, et al., Oxidized LDL, statin use, morbidity, and mortality in patients receiving maintenance hemodialysis. Free Radic Res, 2017. 51(1): p. 14-23.
5. Rusu, C.C., S. Racasan, I.M. Kacso, D. Moldovan, A. Potra, I.M. Patiu, D. Vladutiu, and M.G. Caprioara, Malondialdehyde can predict survival in hemodialysis patients. Clujul Med, 2016. 89(2): p. 250-6.
6. Hsiao, K.C., J.P. Tsai, S.F. Yang, W.C. Lee, J.Y. Huang, S.C. Chang, C.S. Hso, and H.R. Chang, MMP-2 serum concentrations predict mortality in hemodialysis patients: a 5-year cohort study. Clin Chim Acta, 2016. 452: p. 161-6.
7. Lee, Y.K., D.H. Lee, J.K. Kim, M.J. Park, J.J. Yan, D.K. Song, N.D. Vaziri, and J.W. Noh, Lysophosphatidylcholine, oxidized low-density lipoprotein and cardiovascular disease in Korean hemodialysis patients: analysis at 5 years of follow-up. J Korean Med Sci, 2013. 28(2): p. 268-73.
8. Sevinc Ok, E., F. Kircelli, G. Asci, E. Altunel, M. Ertilav, S. Sipahi, D. Bozkurt, S. Duman, M. Ozkahya, H. Toz, et al., Neither oxidized nor anti-oxidized low-density lipoprotein level is associated with atherosclerosis or mortality in hemodialysis patients. Hemodial Int, 2012. 16(3): p. 334-41.
9. Wang, A.Y., C.W. Lam, I.H. Chan, M. Wang, S.F. Lui, and J.E. Sanderson, Prognostic value of plasma myeloperoxidase in ESRD patients. Am J Kidney Dis, 2010. 56(5): p. 937-46.
10. Pachaly, M.A., M.M. do Nascimento, M.E. Suliman, S.Y. Hayashi, M.C. Riella, R.C. Manfro, P. Stenvinkel, and B. Lindholm, Interleukin-6 is a better predictor of mortality as compared to C-reactive protein, homocysteine, pentosidine and advanced oxidation protein products in hemodialysis patients. Blood Purif, 2008. 26(2): p. 204-10.
11. Kalantar-Zadeh, K., M.L. Brennan, and S.L. Hazen, Serum myeloperoxidase and mortality in maintenance hemodialysis patients. Am J Kidney Dis, 2006. 48(1): p. 59-68.
